# Effectiveness of an innovative system for the bio-decontamination of the ICU

**DOI:** 10.1186/cc10687

**Published:** 2012-03-20

**Authors:** A De Nicola, MJ Sucre

**Affiliations:** 1 San Leonardo Hospital, Castellammare di Stabia, Italy

## Introduction

The ICU contains a large quantity of sensitive electrical equipment which must not be affected by any bio-decontamination process. To disinfect the ICU environment we have used the original device, Medisize 99.99^®^, which releases a synergistic formulation of hydrogen peroxide with silver ions. The machine launches a dry cloud of 0.5 to 2 m particles which penetrates everywhere, without humidity or corrosive activity.

## Methods

The study has been conducted in the ICU area, just after the patients have been discharged, before and after the use of the Medisize 99.99^® ^device. The overall number of samples taken has been 54 on three different days. The sampling has been taken with Petri contact plates and incubated at 35°C for 48 hours, counting afterwards the CFU/plate.

## Results

We found the annulment of the contamination at all sites tested after sanitation (Table 1).

**Table 1:** CFU of sample sites before and after sanitation

Sample sites	Before UFC/cm^2^	After UFC/cm^2^
Mattress	10^4^	<0.5
Vital parameters monitor	5,000	<0.5
Wall	2,000	<0.5
Bed rail	<0.5	<0.5
Bed remote control	<0.5	<0.5
Ventilator screen	5,000	<0.5
Ventilator chassis	10^4^	<0.5
Infusion pump	<0.5	<0.5

## Conclusion

The destruction of the bacteria has practically taken place in all the points tested. The system has resulted to be compatible with the electronic equipment and a few minutes after the end of the procedure it is possible to use the area. The catalytic action of the silver atoms produces the tyndallisation of the surfaces, increasing the effectiveness of the sanitizer. Eight minutes after the end of the treatment, 98% of the OH^- ^radicals have been destroyed and 95% of the dry cloud has been deposited, inhibiting the possibility of regeneration of any resistant microorganism.

